# Successful conservative treatment of MRSA-associated infective endocarditis with concurrent candidemia in a hemodialysis patient

**DOI:** 10.1186/s43044-026-00754-3

**Published:** 2026-06-03

**Authors:** Ömer Faruk Yilmaz, Oğuzhan Dilek, Ömer Kutsi Misirlioğlu, Tuba Bozoklu, Havva Tünay, Serkan Gökaslan, Neşe Demirtürk

**Affiliations:** 1 Cardiology, Kaman State Hospital, Kırşehir, Turkey; 2 Infectious Diseases, Afyonkarahisar State Hospital, Afyonkarahisar, Turkey; 3 Cardiology, Afyonkarahisar Health Sciences University, Afyonkarahisar, Turkey; 4 Infectious Diseases, Afyonkarahisar Health Sciences University, Afyonkarahisar, Turkey

**Keywords:** Candidemia, Chronic kidney disease, Hemodialysis, Infective endocarditis, Methicillin-resistant *Staphylococcus aureus*

## Abstract

**Background:**

Infective endocarditis carries high mortality in hemodialysis patients, particularly when caused by methicillin‑resistant *Staphylococcus aureus*. Concomitant fungemia further worsens prognosis but remains rare.

**Case summary:**

A 52‑year‑old woman with type 2 diabetes mellitus and stage V chronic kidney disease on thrice‑weekly hemodialysis presented with atypical chest pain, fatigue, night sweats, and splinter hemorrhages. She was afebrile. Transthoracic echocardiography showed preserved left‑ventricular ejection fraction and grade II mitral/tricuspid regurgitation; transesophageal echocardiography revealed a 0.7 × 2.1 cm pedunculated vegetation on the septal leaflet of the tricuspid valve. Three sets of peripheral and catheter blood cultures were drawn, and empiric renally adjusted daptomycin plus gentamicin were started. On day 3, C‑reactive protein had fallen. Cultures grew methicillin‑resistant *Staphylococcus aureus* in two peripheral sets and in the tunneled catheter, and Candida tropicalis in one peripheral set. The infected catheter was removed and a brachio‑axillary graft was placed. Gentamicin was discontinued; daptomycin was continued for six weeks. Voriconazole was administered for 21 days, ending 14 days after negative fungal cultures. Follow‑up echocardiography at week 6 showed complete resolution of the vegetation. The patient remained asymptomatic at three‑month follow‑up.

**Conclusion:**

In immunocompromised hemodialysis patients, dual bacterial‑fungal bloodstream infections can occur, yet not every positive fungal culture indicates endocardial involvement. Serial cultures, prompt removal of infected hardware, and targeted antimicrobial therapy can achieve cure without surgery even in the presence of sizable vegetations.

## Background

 Infective endocarditis (IE) is a severe cardiovascular infection of the heart valves or endocardium caused by bacteria, fungi, or other microorganisms. The incidence of IE in the general population is estimated to be 13.7 cases per 100.000 person-years [1, 2]. Among hemodialysis (HD) patients, the prevalence is estimated at 2.9% (95% confidence interval [CI] 2.7–3.1%), with significant morbidity and mortality rates reported [3]. Congenital heart disease, prosthetic heart valves, intravenous (IV) drug use, and HD increase the risk of IE. Streptococcus and Staphylococcus species are the most common pathogens involved. However, methicillin-resistant *Staphylococcus aureus* (MRSA) and Candida species are also rare but possible pathogens in IE [4].

The primary clinical manifestations of IE include fever, fatigue, night sweats, anorexia, weight loss, joint pains, and chest pain. Physical examination may reveal splenomegaly, petechiae, Osler nodes, Janeway lesions, and splinter hemorrhages [4]. Diagnosis is typically confirmed through transthoracic or transesophageal echocardiography (ECHO) and blood cultures for pathogen isolation. Treatment involves antimicrobial therapy, primarily antibiotics, and surgical intervention in selected cases. Prompt diagnosis and appropriate antimicrobial therapy are critical for successful management due to the potential for heart failure, valve dysfunction, and systemic embolization resulting from delayed treatment [2]. This case report presents an IE caused by MRSA with concomitant candidemia in a patient undergoing HD for diabetes mellitus (DM) and chronic kidney disease (CKD). The patient’s early diagnosis, rapid response to antibiotic therapy without surgical intervention, and the presence of concomitant candidemia highlight the risk of IE in HD patients.

## Case presentation

**Fig. 1:**
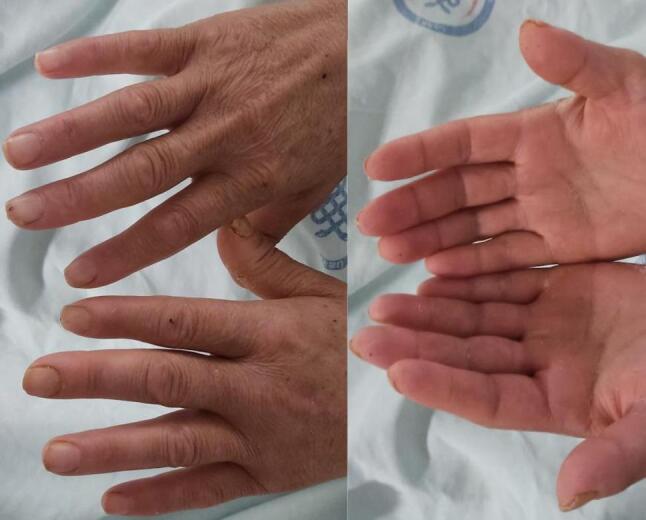
Suspicious splinter hemorrhages on the patient’s fingernails; no Osler nodules or Janeway lesions were found

A 52-year-old female patient with long-standing DM and stage V CKD receiving maintenance HD was initially hospitalized in the internal medicine/nephrology unit for diabetic ketoacidosis after missing her basal insulin dose. The exact primary etiology of CKD was not definitively documented in the available medical records. During this admission, her plasma glucose level had increased to approximately 750 mg/dL and subsequently decreased to approximately 350 mg/dL with treatment. Urinalysis showed positive ketones, and blood gas analysis demonstrated metabolic acidosis with a pH of 7.29 and bicarbonate level of 19.0 mmol/L. She was managed with insulin infusion, hydration, and close metabolic monitoring. During follow-up, chest pain developed and was accompanied by progression of cardiac biomarkers, prompting transfer to the cardiology clinic for further evaluation. Initial cardiology assessment showed no fever or dyspnea but revealed fatigue, night sweats, mild abdominal pain, and chest pain. Although the chest pain was initially evaluated in the context of acute coronary syndrome, the presence of night sweats, a systolic murmur, and suspicious splinter hemorrhage-like lesions on the fingernails also raised concern for concomitant infective endocarditis. No Osler nodes, Janeway lesions, or petechiae were found (Fig. 1).

**Fig. 2:**
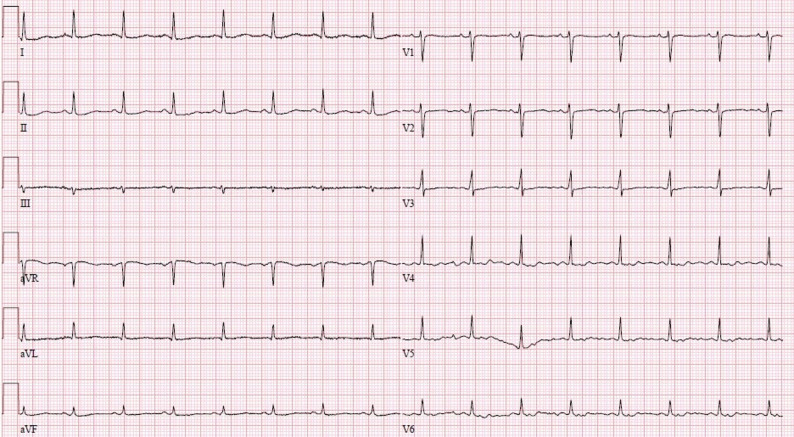
The patient’s pre-chest pain ECG showing sinus rhythm and no pathology in the ST-T waves

**Fig. 3:**
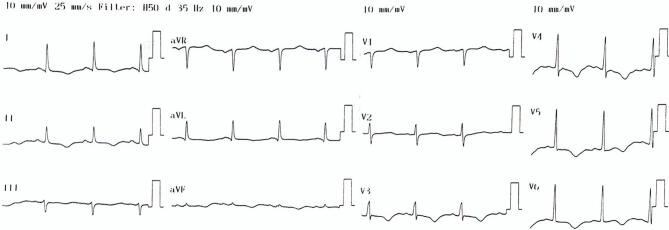
The patient’s post-chest pain ECG showing sinus rhythm, a heart rate of 86 bpm, and T wave inversions in D1, D2, aVL, and V3-6 leads, indicating dynamic changes compared to previous ECGs

Cardiac examination revealed a 2/6 systolic murmur at the mitral and tricuspid areas. Basal inspiratory crackles were heard in the lower regions during lung auscultation. Vital signs were stable with no fever. Serial clinical assessments were performed to monitor for potential embolic complications. Arterial and venous Doppler ultrasonography of the upper and lower extremities showed patent vascular flow without evidence of thrombus or embolic occlusion. Computed tomography angiography of the thorax, abdomen, and pelvis, including pulmonary arterial evaluation, revealed no evidence of pulmonary embolism, intra-abdominal thrombus, or other radiologically apparent embolic complication. Neurological examinations remained normal throughout hospitalization, with no focal neurological deficit, altered mental status, or seizure. Electrocardiography (ECG) showed a sinus rhythm with a heart rate of 86 beats per minute and T wave inversions in D1, D2, aVL, and V3-6 leads, without ST segment elevation. Her ECG showed dynamic changes compared to her previous ECGs (Figs. 2 and 3).

**Fig. 4:**
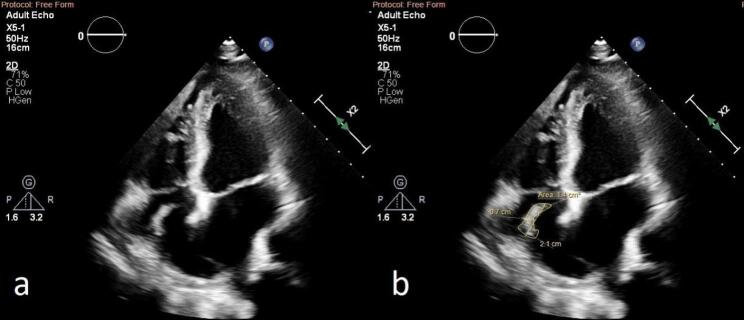
**a** Vegetation in the right atrium extending to the tricuspid valve. **b** The vegetation measured 0.7 × 2.1 cm

Laboratory results showed stable renal function and normal electrolyte levels, with no significant elevation of inflammatory markers at admission. Pro-BNP was available only at the initial assessment and was elevated at 10,567 ng/L. Given the patient’s advanced CKD and maintenance HD status, this single natriuretic peptide value was interpreted cautiously and was not used as a serial marker of treatment response. Subsequent clinical monitoring was guided by symptoms, physical examination, echocardiographic findings, blood cultures, inflammatory markers, and cardiac biomarkers. Follow-up measurements of cardiac biomarkers demonstrated progression (Table 1). ECHO revealed a normal left ventricular ejection fraction and no segmental wall motion abnormality but detected grade II mitral and tricuspid regurgitation. Transesophageal echocardiography confirmed a suspicious vegetation measuring 0.7 × 2.1 cm extending toward the tricuspid valve (Fig. 4).

**Table 1:** Laboratory findings during hospitalization

Name of blood test	First examination	Follow-up	Day 3	Day 8	Week 6	Reference values
Urea (mg/dL)	61.4	-	-	-	-	16.6–48.5
Bun (mg/dL)	28.69	-	-	-	-	7–22
Creatinine (mg/dL)	2.52	-	-	-	-	0.5–0.9
eGFR (mL/min/1.73 m²) (CKD-EPI Calculation)	16	-	-	-	-	> 90
Sodium (mmol/L)	135	-	-	-	-	135–145
Potassium (mmol/L)	4.2	-	-	-	-	3.5–5.1
Chlorine (mmol/L)	95	-	-	-	-	97–111
Calcium (mg/dL)	8.40	-	-	-	-	8.6–10.0
Phosphor (mg/dL)	4.79	-	-	-	-	2.5–4.5
C-Reactive Protein (mg/L)	4.2	-	101.3	75.8	4.5	0–5.0
WBC (10^3/uL)	12.00	-	8.49	6.53	6.27	4–10
NEU# (10^3/uL)	10.85	-	7.18	4.59	4.01	1.5–6
LY# (10^3/uL)	0.75	-	0.19	0.67	1.21	1.2–4
MO# (10^3/uL)	0.39	-	0.3	0.89	0.63	0.2–0.8
BA# (10^3/uL)	0.01	-	0.03	0.01	0.05	0–0.1
EO# (10^3/uL)	0.00	-	0.79	0.37	0.37	0–0.4
HGB (g/dl)	9.3	-	10.1	8.6	8.2	12–16
PLT (10^3/uL)	218	-	88	102	92	160–370
Pro-BNP (ng/L)	10,567	-	-	-	-	0–125
D-Dimer (µg FEU/mL)	0.52	-	-	-	0.56	0–0.5
CK (IU/L)	94	120	-	-	24	0–190
CK-MB mass (µg/L)	4.66	8.10	-	-	1.62	1.39–4.88
hs-Troponin T (ng/mL)	0.158	0.394	-	-	0.058	0–0.014

Table depicts the initial laboratory blood tests conducted upon the patient’s admission to the cardiology clinic, as well as subsequent evaluations of cardiac markers and acute phase reactants during the course of their hospitalization

**Fig. 5:**
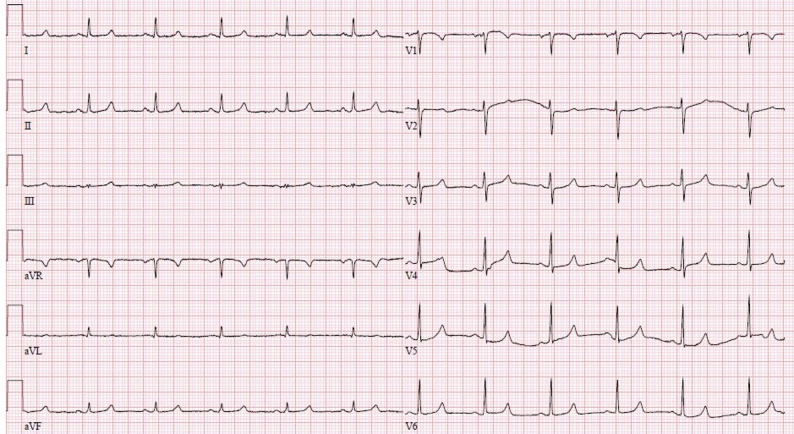
The patient’s ECG one month after LAD stent implantation showing resolution of T wave inversions

Coronary angiography performed for non-ST elevation myocardial infarction (NSTEMI) revealed a critical lesion in the left anterior descending (LAD) coronary artery, which was treated with stent implantation. A follow-up ECG performed one month after LAD stent implantation demonstrated resolution of the previously observed T-wave inversions (Fig. 5).

Meanwhile, for the evaluation of infective endocarditis (IE) etiology, aerobic and anaerobic blood cultures were obtained from peripheral sites and from the permanent HD catheter. As part of the etiological assessment, the patient was also questioned for potential extracardiac sources of bacteremia; there was no recent history of tooth extraction or invasive dental procedure. After obtaining three sets of blood cultures, empiric antibiotic therapy was initiated with renal-adjusted doses of daptomycin (6 mg/kg IV every 48 h) and gentamicin (2 mg/kg IV every 48 h) due to suspicion of healthcare-associated IE. On the third day of antibiotic therapy, a regression in C-reactive protein (CRP) was observed. MRSA was isolated from two peripheral blood cultures and from the permanent HD catheter culture, whereas Candida tropicalis was isolated from a single peripheral blood culture.

Blood samples were processed using the BACTEC FX automated blood culture system (Becton Dickinson, USA). Bottles signaling microbial growth were subcultured onto routine microbiological media and incubated at 37 °C for 18–24 h. Yeast colonies were initially evaluated by Gram staining and conventional methods including the germ tube test. Species-level identification and antifungal susceptibility testing were performed using the VITEK 2 Compact automated system (bioMérieux, France) with the yeast identification card (YST) and the corresponding AST susceptibility card. Susceptibility results were interpreted according to the current European Committee on Antimicrobial Susceptibility Testing (EUCAST) breakpoint tables for Candida species.

**Fig. 6:**
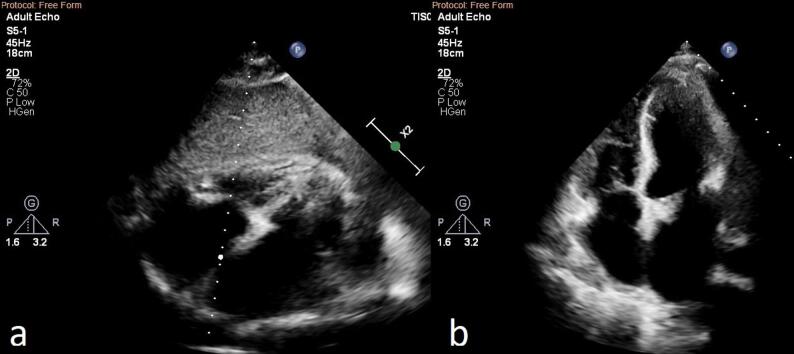
**a** Subxiphoid echocardiographic window after 6 weeks of antibiotic therapy showing no vegetation/mass. **b** Apical echocardiographic window showing no vegetation/mass

Candida tropicalis was isolated from a single peripheral blood culture, whereas subsequent blood cultures remained negative. Ophthalmologic examination was performed and no evidence of Candida endophthalmitis or other ocular involvement was detected. Serum β-D-glucan testing was not available in our center; therefore, the diagnostic assessment relied on serial blood cultures, clinical evaluation, and echocardiographic findings. Following these findings, the permanent HD catheter implanted in the right innominate vein seven months earlier was removed as a potential source of infection. To continue HD, a chemical-bonded dialysis graft was created between the left brachial artery and axillary vein due to the graft’s superiority and inability to create a fistula from the patient’s own vessel. Antibiotic therapy was continued according to MRSA sensitivity. Based on microbiological and clinical findings, MRSA was considered the primary causative pathogen of infective endocarditis. Nevertheless, given the isolation of Candida tropicalis, antifungal therapy with intravenous voriconazole was initiated with a loading dose of 6 mg/kg followed by a maintenance dose of 4 mg/kg. Treatment was continued for a total of 21 days, including 14 days after the first negative blood culture. Gentamicin was discontinued, and daptomycin therapy was extended to 6 weeks. Follow-up tests showed negative acute phase reactants, regressed cardiac markers, no growth in blood cultures, and no vegetation on follow-up ECHO (Fig. 6). The patient was discharged in good clinical condition with no signs of infection or cardiac symptoms and remained free of additional cardiac pathology during follow-up.

## Discussion

This article presents a rare case of MRSA-associated IE with concomitant candidemia. Infective endocarditis is usually associated with bacterial pathogens, with MRSA being among them [4]. However, MRSA-associated IE cases with concomitant candidemia are quite rare. In this diabetic patient with an HD catheter, IE was incidentally detected. Candida and MRSA grew in blood cultures, but Candida was not identified as the IE pathogen. Appropriate medical treatment allowed clinical recovery without the need for surgical intervention. Although the tricuspid vegetation was relatively large, vegetation size alone is not regarded as an isolated indication for surgery in right-sided infective endocarditis. Current ESC guidelines recommend surgery for right-sided infective endocarditis mainly in the presence of persistent bacteremia or sepsis despite appropriate antimicrobial therapy, severe tricuspid regurgitation with right ventricular dysfunction refractory to diuretic therapy, respiratory insufficiency or large residual tricuspid vegetations after recurrent septic pulmonary emboli, or concomitant left-sided involvement [2]. In the present case, the absence of persistent bacteremia, severe right-sided heart failure, respiratory compromise, or documented left-sided valve involvement, together with clinical and echocardiographic improvement under targeted antimicrobial therapy, supported an individualized conservative strategy with close follow-up.

IE poses a significant risk of high mortality and morbidity. Underlying predisposing factors, inappropriate antimicrobial therapy, and delayed diagnosis negatively impact outcomes [2, 5]. While staphylococci and streptococci are commonly identified in the etiology, fungal pathogens, though rare, can also be observed. Candida albicans is the most common fungal agent in IE, but non-albicans Candida species and Aspergillus can also be etiological agents [2, 6]. In recent years, an increase in MRSA and fungal pathogens has been observed, particularly among immunosuppressed and HD patients [5]. In the study by Nirmanmoh Bhatia et al. [7], approximately 2% of chronic HD patients experienced IE, which is similar to the 2.9% incidence rate in HD patients in the meta-analysis of 18 studies by Masoumeh Sadeghi et al. Additionally, Nirmanmoh Bhatia et al. [7] reported higher mortality in HD patients compared to non-HD patients (aOR: 1.89, 95% CI: 1.80–1.99, *P* < 0.001). Microbiological pathogens in HD patients’ blood cultures include staphylococci (most commonly *Staphylococcus aureus* at 75.5%), streptococci (14%), gram-negative bacilli (10.2%), enterococci (8.8%), and fungi (0.4%). Compared to non-HD control groups, staphylococci and fungal pathogens were more common in the HD group. Our patient had significant underlying risk factors such as DM and CKD, with MRSA playing a role in the etiology. Additionally, Candida tropicalis growth was observed in blood cultures, indicating a high risk for mortality and morbidity, leading to intensive care monitoring.

The frequency and treatment of pathogens in IE etiology vary depending on valve structure. Since our patient had a natural valve, we focused on natural valve IE. Staphylococci are involved in 11% of natural valve IE cases, often coagulase-negative staphylococci (CNS), with MRSA being rare. CNS infections can sometimes be confused with Staphylococcus lugdunensis [6]. In our case, MRSA was identified in two different blood cultures and catheter cultures, differentiated from Staphylococcus lugdunensis by coagulase positivity. MRSA infections, due to increasing antibiotic resistance, pose serious challenges and have high mortality rates, necessitating aggressive antimicrobial therapy [6]. Anthony D Bai et al. [8] reported a 27% (95% CI 21.5–33.3%) three-month mortality rate in patients with *Staphylococcus aureus* bacteremia, with higher mortality in methicillin-resistant cases (adjusted OR [aOR]: 1.04; 95% CI 1.02–1.06 per 10% increase in methicillin-resistant S. aureus ratio). Our patient responded quickly to empirical Daptomycin treatment, which was continued after MRSA growth was detected.

However, the concomitant isolation of Candida tropicalis from a single peripheral blood culture required careful interpretation. The isolate was considered clinically significant rather than dismissed as contamination, because Candida species recovered from blood cultures generally warrant prompt clinical attention, particularly in high-risk patients with diabetes, chronic kidney disease, and indwelling vascular catheters [9–12]. Therefore, the central diagnostic question in this case was not whether Candida tropicalis represented contamination, but whether it contributed to the pathogenesis of infective endocarditis. In the present case, several findings supported true but transient candidemia without definite evidence of fungal infective endocarditis. First, Candida tropicalis was isolated from only a single peripheral blood culture, without persistent fungemia. Second, follow-up blood cultures remained consistently negative after catheter removal. Third, transesophageal echocardiography did not reveal findings suggestive of large or atypical vegetations typically associated with fungal endocarditis, although echocardiography alone is insufficient to definitively exclude fungal involvement. Finally, there was no ocular involvement or clinically documented metastatic complication. Serum β-D-glucan testing was not available in our center; therefore, the diagnostic assessment relied on serial blood cultures, echocardiographic findings, ophthalmologic examination, and clinical response. In accordance with candidemia management recommendations, the hemodialysis catheter was removed, follow-up blood cultures were obtained, ophthalmologic examination was performed, and antifungal therapy was continued for 14 days after documented blood-culture clearance, resulting in a total treatment duration of 21 days [6, 9]. These findings underscore that Candida isolated from blood culture should be managed as clinically significant candidemia, while also highlighting that not every episode of candidemia necessarily indicates fungal involvement of the endocardium.

The permanent HD catheter was removed due to being a source of infection, and a new access route was established. According to literature-based consensus recommendations, the permanent catheter was removed, and a chemical-bonded dialysis graft was applied due to its superiority over fistula and HD catheter [6].

In conclusion, atypical pathogens such as MRSA and fungi should be considered in IE cases developing in immunosuppressed or dialysis patients, and appropriate antimicrobial therapy should be determined. Control of underlying risk factors, early diagnosis, and aggressive treatment are critical in managing this severe infection.

## Data Availability

No datasets were generated or analysed during the current study.

## Abbreviations

CI: Confidence interval
CKD: Chronic kidney disease
CNS: Coagulase negative staphylococci
CRP: C-reactive protein
DM: Diabetes mellitus
ECG: Electrocardiography
ECHO: Echocardiography
EUCAST: European Committee on Antimicrobial Susceptibility Testing
HD: Hemodialysis
IE: Infective endocarditis
IV: Intravenous
LAD: Left anterior descending
MRSA: Methicillin-resistant *Staphylococcus aureus*
NSTEMI: Non-ST elevation myocardial infarction
YST: Yeast identification card
